# Overexpression of hsa_circ_0002874 promotes resistance of non-small cell lung cancer to paclitaxel by modulating miR-1273f/MDM2/p53 pathway

**DOI:** 10.18632/aging.202521

**Published:** 2021-02-17

**Authors:** Jianhao Xu, Liwei Ni, Fenglun Zhao, Xiaoxiao Dai, Jialong Tao, Jia Pan, Aiming Shi, Zhu Shen, Cunjin Su, Yusong Zhang

**Affiliations:** 1 Department of Pathology, Kunshan First People’s Hospital Affiliated to Jiangsu University, Kunshan 215300, Jiangsu, PR China; 2 Department of Medical Oncology, Hangzhou Cancer Hospital, Hangzhou 310002, Zhejiang, PR China; 3 Department of Pharmacy, The Second Affiliated Hospital of Soochow University, Suzhou 215004, Jiangsu, PR China; 4 Department of Pathology, The Second Affiliated Hospital of Soochow University, Suzhou 215004, Jiangsu, PR China; 5 Department of Oncology, The Second Affiliated Hospital of Soochow University, Suzhou 215004, Jiangsu, PR China

**Keywords:** hsa_circ_0002874, miR1273f, MDM2, P53, paclitaxel-resistance

## Abstract

Background: This study aimed to investigate the aberrant expression of hsa_circ_0002874 in non-small cell lung cancer (NSCLC) and elucidate associated molecular mechanisms that influence apoptosis and induce paclitaxel (PTX) resistance.

Methods: Inhibitors were used to downregulate circRNA or miRNA expression. pCDNA plasmid transfection and mimics were used to upregulate circRNA or miRNA expression. Dual-luciferase reporter assays were conducted to evaluate interactions between miR1273f and MDM2. Xenograft tumor models were used to assess the effect of hsa_circ_0002874 and miR1273f on tumor growth. NSCLC tissues and matched non-cancerous tissues were also collected for correlation analysis.

Results: hsa_circ_0002874 acts as a sponge for miR1273f which targets MDM2/P53. The stability of the hsa_circ_0002874/miR1273f/MDM2/P53 pathway was verified by upregulating and downregulating the expression of hsa_circ_0002874 and miR1273f. hsa_circ_0002874 downregulation or miR1273f upregulation reversed the resistance of the A549/Taxol cells in xenograft models. The expression of hsa_circ_0002874 was high, and the level of MDM2 was low in NSCLC tissues. P53 was only weakly expressed in NSCLC tissues with high expression of MDM2.

Conclusions: hsa_circ_0002874 is strongly expressed in NSCLC tissues and maybe a potential marker for PTX resistance. hsa_circ_0002874 downregulation could regulate miR1273f/MDM2/P53 signaling pathway to reverse the PTX resistance of NSCLC and induce apoptosis *in vitro* and *vivo*.

## INTRODUCTION

Lung cancer (LC) is one of the most common causes of cancer-related death worldwide [1–3] and ranked second and first among new cancer cases and cancer-related death in 2018, respectively [4, 5]. Non-small cell LC (NSCLC) accounts for 80% of all LC cases with a 5-year survival rate of approximately 10%–15% [6, 7]. For patients with advanced NSCLC who do not receive molecular targeted therapy or immune checkpoint therapy, the standard first-line treatment remains cytotoxic chemotherapy [8]. Paclitaxel (PTX) is an important first-line treatment of advanced NSCLC [9] and interferes with cell division by promoting microtubule polymerization and apoptosis [10]. However, other anti-tumor mechanisms for PTX remain undiscovered. One shortcoming of PTX is the emergence of drug resistance, but the underlying molecular mechanism still under investigation.

Unlike linear RNA, circular RNA (circRNA) forms a covalently closed continuous loop. That is, the 3' and 5' ends usually present in RNA molecules are connected in circular RNA [11]. In molecular biology, competition for endogenous RNA (ceRNA) is based on the function of miRNA to regulate other RNA transcripts [12, 13]. The circRNA hsa_circ_0002874 has been reported to be closely associated with doxorubicin resistance in the breast cancer cell line MCF-7 [14]. It was predicted by the software circMir 1.0 and RegRNA that its target miRNA is miR1273f. However, the miR-1273 family has 33-1074 mRNA target genes (free hybridization energy ≥90%), of which miR-1273f alone has >400 target genes, playing an important role in many different molecular pathways [15].

In this study, we investigated the aberrant expression of hsa_circ_0002874 in NSCLC and elucidated the molecular mechanisms underlying its influence on apoptosis and PTX resistance induction. Our findings provide novel viewpoints for the anti-tumor mechanisms of PTX and the molecular mechanism of PTX resistance in NSCLC.

## RESULTS

### In A549 cells, PTX treatment downregulates the expression of hsa_circ_0002874 which is predicted to act as a sponge for miR1273f

We designed primers for 18 circRNAs based on the reported microarray results from the doxorubicin-resistant breast cancer cell line MCF-7 [14] (Table 1). Screening by qPCR identified circRNA hsa_circ_0002874 as having the largest and most reproducible change of expression level after PTX exposure. Its target miRNAs were predicted by circMir 1.0, RegRNA 2.0 and MirTrap, and target genes of these screened miRNAs were predicted by literature review and miRBase webpage analysis. These were then confirmed by qPCR after PTX administration.

**Table 1:** Primer sequences used for qPCR.

**Gene**	**Forward primer sequence 5'-3'**	**Reverse primer sequence 5'-3'**
hsa_circ_0002113	ACAGATAGAAGAGCACCGACAGT	AGCTCGAAGGCGTAGAGTGA
hsa_circ_0001667	TGCAGGAAACACTGATGCCC	AGGGCAGGGCCAGGATAAAT
hsa_circ_0006528	CGGAGTCACTGCCTTACGTG	AGCAGCTACTGTGTTCACGC
hsa_circ_0002874	TGGAGGCATGTCAGGGTCAC	GTGGGTTATAAGCCTTTCCCAGG
hsa_circ_0002168	CTGCATCACCACAGTTGCAGG	CGGAAGGGTCGGATGAAAGC
hsa_circ_0086241	GGGATTCAGATGGGCGTCAC	TTGTTGTTCGGTGTCGCTGG
hsa_circ_0007769	GCCGACGAACAGAACCACTC	AGAGGAGCCAGCATTTTGCAT
hsa_circ_0092276	CCTAGGAACCTTGTGCTTGCC	CAACCACACACTCCAAGCTCC
hsa_circ_0044556	TGCCAAGGGTCTGACTGGAA	AGGGGGTCCTTGAACACCAA
hsa_circ_0003183	AGGTGAAGCTTTTGCACGAGA	TCTTGCCCTGCCTCTTCTACA
hsa_circ_0085567	GAGGGAACGCCAATCCAAGG	AAATGAATTACCTTCAGCCAGTGC
hsa_circ_0085495	TTGCAGGCTACGTTGAAGCA	AGCCAAACCCTATGAAAGGTATCG
hsa_circ_0008131	AAGAAGGCGTTCGATGCTCC	CAACCTGCGAGGTGGACATT
hsa_circ_0003838	CTGCAATTGGCCTCGAGCTG	CCTGTTCCGATGTGCGTTGA
hsa_circ_0007551	AAGGCATCGACTGGACCCC	GAGCTTTGGGAAGCGGTCAC
hsa_circ_0005004	AGGCAGCTGATGAGGTTTGAG	GATGGTCTTGAGGGCAGGGA
hsa_circ_0006903	ACCACGTCTGGCAGAAGATTT	CAAAGCCTCTTTCCGGGTCC
hsa_circ_0018293	GCAGGCGAGAAGATTCGTGG	TCCATCTGTGCCACCCCTTA
MDM2	CATTGAACCTTGTGTGATTTGTC	GCAGGGCTTATTCCTTTTCTTTA
hsa-miR-1273f	GCTAACAACCTCCATCTCA	CAGTGCGTGTCGTGGAGT
hsa-miR-4726-5p	CCACTCCGGGACTCCTGGCCCC	GGTGAGGCCCTGAGGACCGGGG
hsa-miR-2115-5p	AGCAGCAGGAGTTTGGAAGCC	TCATCGTCCTCAAACCTTCGG
hsa-miR-4649-5p	CTCCGGGACTCCTGGCCCCTCGCCCT	GAGGCCCTGAGGACCGGGGAGCGGGA
u6	GCGCGTCGTGAAGCGTTC	GTGCAGGGTCCGAGGT

**Figure 1:**
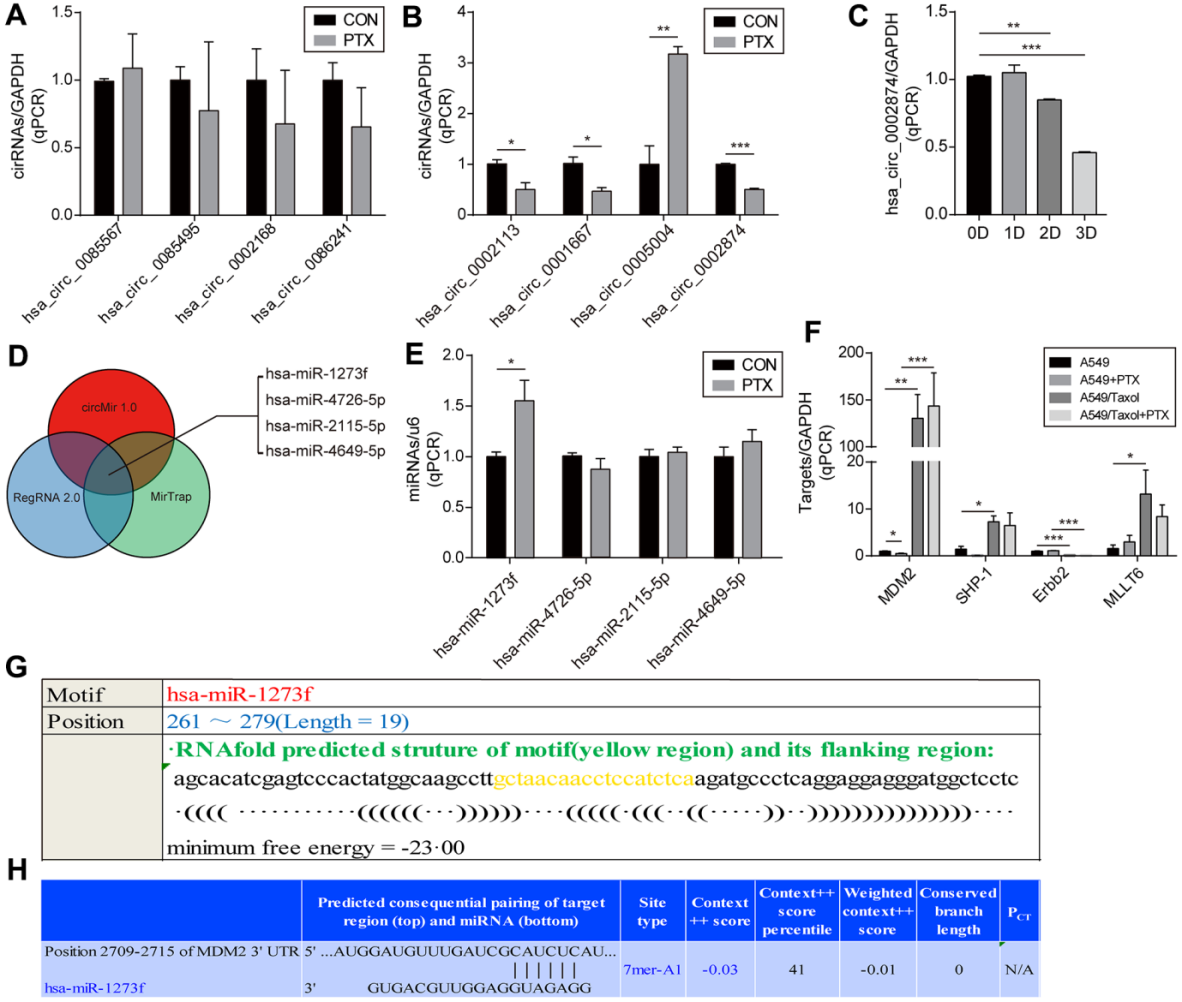
**Screening of circRNA and determination of the downstream miRNA.** (**A, B**) Screening for circRNA with the greatest change after PTX administration by qPCR. (**C**) Confirmation of the hsa_circ_0002874 expression at different time points after PTX administration by qPCR. (**D**) Prediction of target miRNAs for hsa_circ_0002874 by circMir 1.0, RegRNA 2.0 and MirTrap software. (**E**) Changes in the four predicted miRNAs expression after PTX administration by qPCR. (**F**) Changes in target genes expression for 4 predicted miRNAs after PTX administration by qPCR. (**G**) Prediction of the binding stability of hsa_circ_0002874 and miR1273f by Reg RNA2.0 website. (**H**) Prediction of the binding stability of miR1273f and MDM2 mRNA by TargetScan Human 7.2 website. ^*^p <0.05, ^**^p <0.01, ^***^p <0.001, n=3 in each group.

To accomplish this, we first excluded candidates with cycle threshold values (Ct value) of circRNA >30, which is considered too small when the Ct value of GAPDH is 18-20. Among the 18 circRNAs screened, 10 failed this quality control step, namely, hsa_circ_ 0006528, hsa_circ_ 0007769, hsa_circ_ 0092276, hsa_circ_ 0044556, hsa_circ_0003183, hsa_circ_ 0008131, hsa_circ_ 0003838, hsa_circ_ 0007551, hsa_circ_0006903 and hsa_circ_0018293. As shown in Figure 1A, the expression of 4 of the remaining 8 circRNAs did not change significantly (P≥0.05) after PTX administration, namely, hsa_circ_0002168, hsa_circ_0086241, hsa_circ_ 0085567, and hsa_circ_ 0085495. Significant changes in expression after PTX were observed for hsa_circ_ 0002113 (P=0.031), hsa_circ_0001667 (P=0.021), hsa_circ_0005004 (P=0.005), and hsa_circ_ 0002874 (P<0.001) (Figure 1B). Although the most marked change was seen for hsa_circ_0005004, it was found by the technique of Suzhou Gemma Gene Company that its length was too short (spliced length=238 bp) to ensure that the overexpression plasmid was looped. Therefore, a more robust circRNA hsa_circ_ 0002874 (spliced length=486 bp, P<0.001) was selected as the target circRNA for further study. To verify stability, expression of hsa_circ_0002874 at different times after PTX treatment (10 μM) was analyzed by qPCR. A significant decrease in hsa_circ_0002874 expression was found on the 2nd and 3rd day of PTX exposure (P=0.004, P<0.001, respectively) (Figure 1C).

Next, after identifying the target circRNA, target miRNAs were predicted by circMir 1.0, RegRNA 2.0 and MirTrap as follows: hsa-miR-1273f, hsa-miR-4726-5p, hsa-miR-2115-5p and hsa-miR-4649-5p (Figure 1D). As shown in Figure 1E, after exposure to 10μM PTX for 48 h, only hsa-miR-1273f expression had a significant change (P =0.033).

Their target genes were predicted by reviewing the literature and miRBase and Targetscan web page analysis. They were identified as MDM2, SHP-1, Erbb2, and MLLT6 (Table 2). Changed expression of these putative target genes in PTX-sensitive A549 cells compared with the resistant strain A549/Taxol after PTX exposure was analyzed by qPCR. The expression of MDM2 decreased significantly in A549 cells on treatment with PTX (P=0.026), but its high level in A549/Taxol cells was not affected (Figure 1F).

**Table 2:** Screening of target miRNAs for hsa_circ_0002874.

**Name**	**Position**	**Length**	**Sequence**	**Minimum free energy (kcal/mol)**	**Target**
hsa-miR-1273f	261~279	19	GCTAACAACCTCCATCTCA	-23.00	MDM2
hsa-miR-4726-5p	197~218	22	CCACTCCGGGACTCCTGGCCCC	-27.70	SHP-1
hsa-miR-2115-5p	442~462	21	AGCAGCAGGAGTTTGGAAGCC	-15.02	Erbb2
hsa-miR-4649-5p	200~225	26	CTCCGGGACTCCTGGCCCCTCGCCCT	-28.10	MLLT6

Further bioinformatics prediction analysis was undertaken to explore the combination of hsa_circ_0002874 and hsa-miR-1273f, or miR1273f and MDM2. As shown in Figure 1G, RegRNA 2.0 predicted that hsa-miR-1273f is a putative target of hsa_circ_0002874, and Target Scan Human 7.2 that MDM2 is a putative target of hsa-miR-1273f (Figure 1H).

### hsa_circ_0002874 acts as a sponge for miR1273f, thus regulating the expression of MDM2 and P53 in A549 cells and contributing to PTX resistance

To investigate the hsa_circ_0002874/miR1273f/MDM2/P53 pathway and its association with A549/Taxol PTX resistance, relationships between PTX and hsa_circ_0002874, miR1273f, MDM2, or P53 were respectively verified.

**Figure 2:**
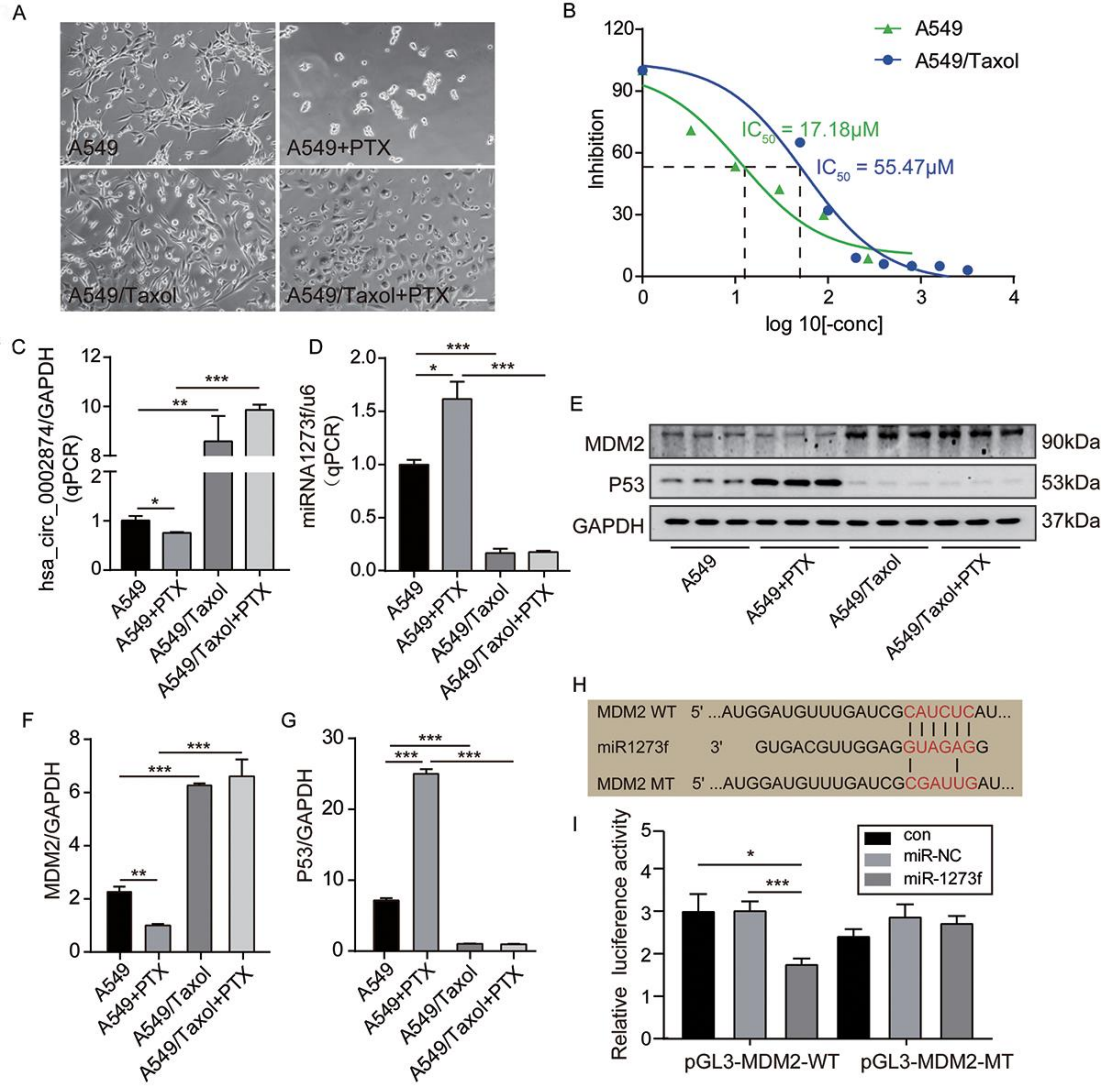
**PTX downregulated the expression of hsa_circ_0002874 and MDM2 while upregulated the expression of miR1273f and P53 in A549 cells.** (**A**) Cell morphology of A549 and A549/Taxol cells following 10μM PTX administration for 48h (images acquired at 10× magnification). (**B**) A549 or A549/Taxol cells viability was measured by MTT assay after the treatment of different concentrations of PTX. (**C**) hsa_circ_0002874 expression was determined by qPCR method after 10μM PTX administration for 48h in A549 and A549/Taxol cells. (**D**) miR1273f expression was determined by qPCR method after 10μM PTX administration for 48h in A549 and A549/Taxol cells. (**E**) MDM2 and P53 expression were determined by western blot after 10μM PTX administration for 48h in A549 and A549/Taxol cells. (**F**) The quantification of MDM2 protein was analyzed according to bands in Figure 2E. (**G**) The quantification of P53 protein was analyzed according to bands in Figure 2E. (**H**) Sequence alignments between miR1273f and the seed sequence of MDM2. WT and MT represent wild-type and mutant sequences of MDM2. (**I**) Results of the dual-luciferase reporter gene assay in 293 cells. *p <0.05, **p <0.01, ***p <0.001, n=3 in each group. Scale bar=100μm.

First, as shown in Figure 2A, after exposure to 10 μM PTX for 48 h, A549 cells appeared smaller and shrunken, whereas no morphological changes of A549/Taxol cells were evident. It was confirmed that PTX inhibited the proliferation of A549 in a dose-dependent manner and that A549/Taxol cells were partially resistant to PTX in this MTT assay (IC_50_ value=17.18 μM and 55.47 μM respectively) (Figure 2B). Intracellular RNA levels were analyzed via qPCR. Expression of hsa_circ_0002874 in A549 cells was significantly decreased after treatment with PTX (P=0.046), while the expression of hsa_circ_0002874 in A549/Taxol cells was very high, about 9-fold that in A549 cells (P=0.002). There was no significant change of hsa_circ_0002874 expression in A549/Taxol cells after PTX treatment, which increased to 15-fold in treated A549 cells (P<0.001) (Figure 2C). Expression of miR1273f was significantly increased in A549 cells treated with PTX (P=0.021), while the expression of miR1273f in A549/Taxol cells was deficient, only one-fifth of that in A549 cells (P=0.002). There was no significant change of miR1273f expression in A549/Taxol cells after PTX treatment, which was one-seventh of that in treated A549 cells (P<0.001) (Figure 2D).

Second, the regulation of MDM2 and P53 levels were analyzed by Western blotting. It was found that the amount of MDM2 protein was significantly decreased in A549 cells after treatment with PTX (P=0.004) (Figure 2E, 2F), while P53 protein was significantly increased (P<0.001) (Figure 2E, 2G). In contrast, the amount of MDM2 protein in A549/Taxol cells was high, almost 3-fold that in A549 cells (P<0.001) (Figure 2E, 2F), while P53 expression was low, about one-eighth of that in A549 cells (P<0.001) (Figure 2E, 2G). There were no significant changes of MDM2 and P53 expression in A549/Taxol cells after PTX treatment, and MDM2 was 6-fold that in treated A549 cells (P<0.001) (Figure 2E, 2F) while P53 was approximately one-twenty-fifth of that in treated A549 (P<0.001) (Figure 2E, 2G).

Third, based on the above results, the negative regulatory activity of miR1273f on MDM2 expression was verified by dual-luciferase reporter gene assays. It was predicted by miRBase and TargetScan that the target gene of miR1273f is MDM2. To test this, the predicted binding sites were mutated (Figure 2H), and a luciferase reporter plasmid containing wild-type or mutant MDM2 was used to observe effects after transfection of MDM2-WT (Wild-type) and MDM2-MT (Mutant-type) (Figure 2I). Luciferase activity was measured 48 hours after transfection. When MDM2-WT was co-transfected together with miR1273f, the relative luciferase activity was significantly lower than the control group or the miR-NC group (P=0.027, P=0.026 respectively) (Figure 2I). However, when co-transfected with MDM2-MT, there was no observable difference. These results indicate that miR1273f interacts with MDM2.

### Knockdown or overexpression of hsa_circ_0002874 regulates the expression of miR1273f, MDM2, and P53 in A549 cells

To further analyze relationships among hsa_circ_0002874, miR1273f, MDM2, and P53, expression of hsa_circ_0002874 was downregulated or upregulated in A549 cells by siRNAs-ciR interference or pCD25-ciR plasmid transfection. siRNAs-ciR is a group of short interfering RNAs used to down-regulate the expression of circular RNAs. pCD25-ciR is the fifth generation circRNA expression vector, which is used to overexpress circRNA. Thereafter, changes in the expression of miR1273f, MDM2 and P53 were analyzed by qPCR and western blotting.

**Figure 3:**
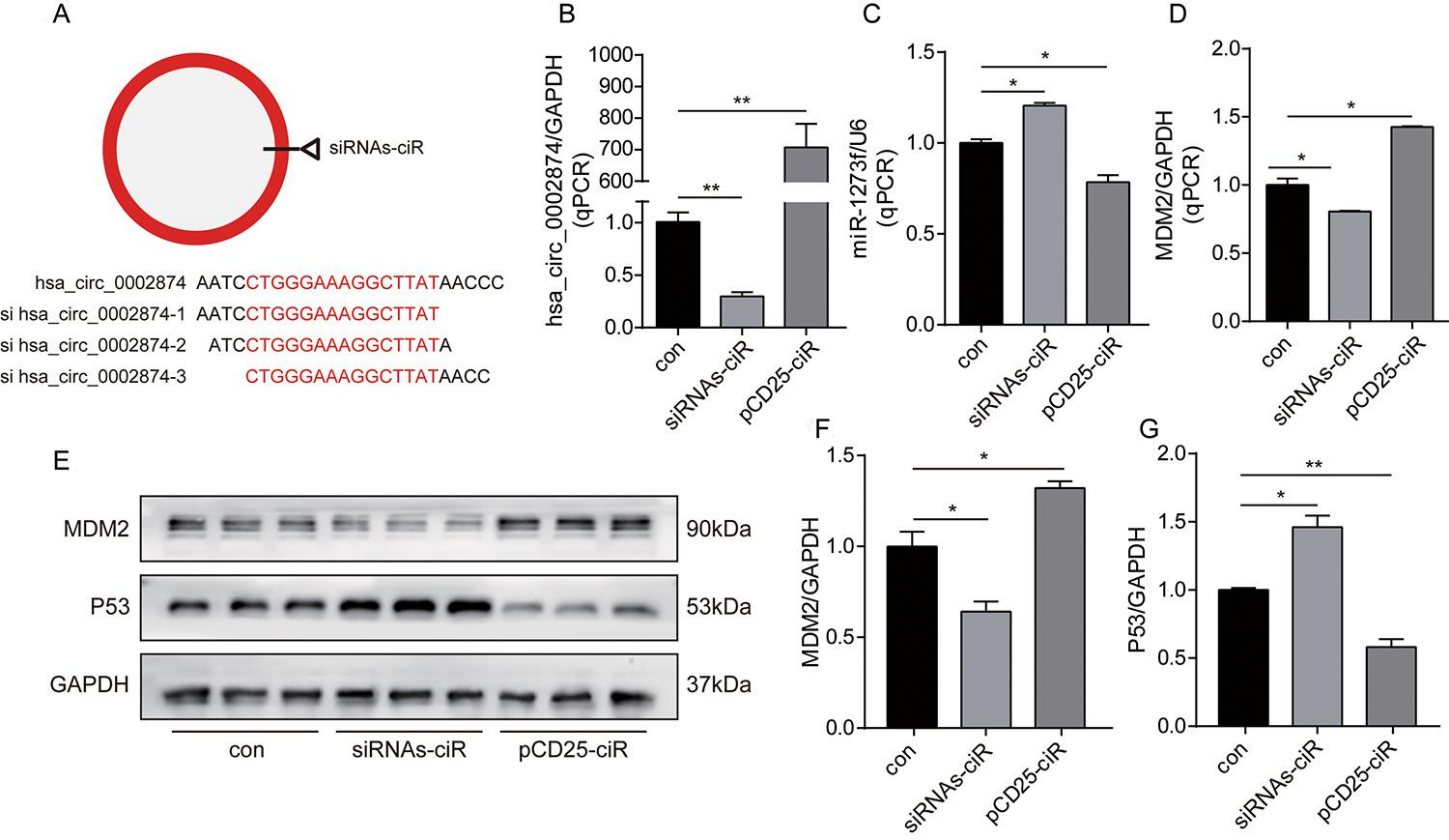
**Knockdown or overexpression of hsa_circ_0002874 regulated the expression of miR1273f, MDM2 and P53 in A549 cell line.** (**A**) 3 hsa_circ_0002874 siRNAs specifically targeting the backsplice junction sequences at different binding sites in hsa_circ_0002874 were designed. (**B**) hsa_circ_0002874 was determined by qPCR method after transfection of siRNAs-ciR and pCD25-ciR. (**C**) miR1273f was determined by qPCR method after transfection of siRNAs-ciR and pCD25-ciR. (**D**) MDM2 mRNA level was determined by qPCR method after transfection of siRNAs-ciR and pCD25-ciR. (**E**) MDM2 and P53 expression were determined by western blot after transfection of siRNAs-ciR and pCD25-ciR. (**F**) The quantification of MDM2 protein was analyzed according to bands in Figure 3E. (**G**) The quantification of P53 protein was analyzed according to bands in Figure 3E. *p <0.05, **p <0.01, ***p <0.001, n=3 in each group.

First, as depicted in Figure 3A, we designed 3 hsa_circ_0002874 siRNAs specifically targeting the back-splice junction sequences at different binding sites in hsa_circ_0002874. As shown in Figure 3B, siRNAs-ciR transfection significantly downregulated the expression of hsa_circ_0002874 to one-third that of the negative control group, as assessed by qPCR (P=0.002), while pCD25-ciR transfection dramatically upregulated the expression of hsa_circ_0002874 to 700 times that in negative control group (P=0.001).

Second, intracellular RNA levels were analyzed via qPCR. As shown in Figure 3C, the expression of miR1273f was upregulated by siRNAs-ciR transfection (P=0.021), while pCD25-ciR transfection significantly decreased it (P=0.039). These results document the negative association of hsa_circ_0002874 with miR1273f. Accordingly, MDM2 mRNA was downregulated by siRNAs-ciR transfection (P=0.044), while it was upregulated by pCD25-ciR transfection (P=0.011) (Figure 3D). These results show that hsa_circ_0002874 is negatively correlated with miR1273f and positively with MDM2.

Next, MDM2 and P53 levels were analyzed by Western blotting. As shown in Figure 3E, siRNAs-ciR transfection downregulated MDM2 expression at the protein level to two-thirds of that in the negative control group (P=0.022) (Figure 3F) and upregulated P53 expression 1.5-fold (P=0.023) (Figure 3G). pCD25-ciR transfection increased MDM2 expression to 1.2-fold that of the negative control (P=0.016) (Figure 3F), and decreased P53 expression by one-half (P=0.006) (Figure 3G). These results indicate that hsa_circ_0002874 expression positively correlates with MDM2 protein levels but negatively with P53.

### Knockdown and overexpression of miR1273f could reduce PTX sensitivity in A549 cell line and reverse PTX resistance in A549/Taxol cell line, respectively

**Figure 4:**
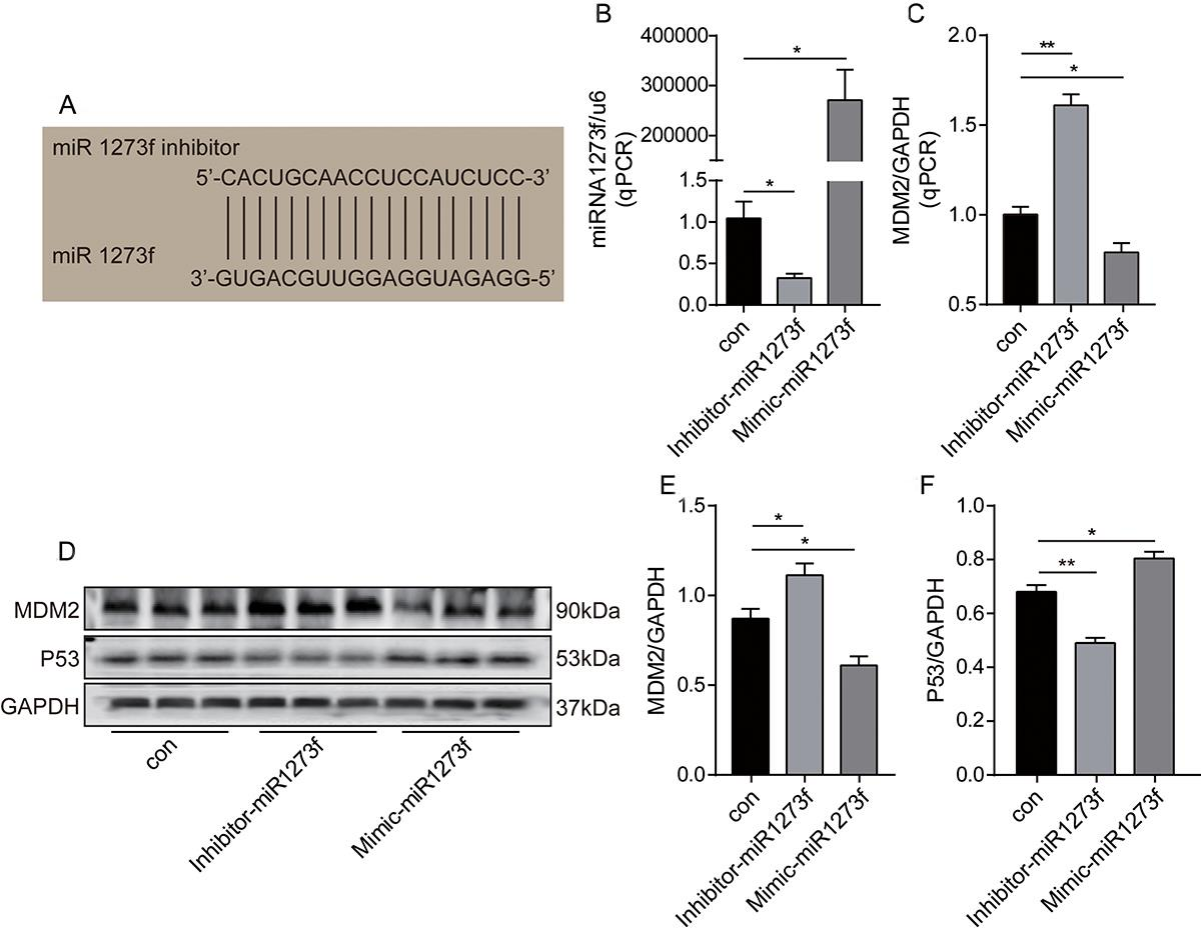
**Knockdown or overexpression of miR1273f regulated the expression of MDM2 and P53 in A549 cell line.** (**A**) miR1273f inhibitor specifically targeting the binding sites in miR1273f was designed. (**B**) miR1273f was determined by qPCR method after transfection of inhibitor-miR1273f and mimic-miR1273f. (**C**) MDM2 mRNA level was determined by qPCR method after transfection of inhibitor-miR1273f and mimic-miR1273f. (**D**) MDM2 and P53 expression were determined by western blot after transfection of inhibitor-miR1273f and mimic-miR1273f. (**E**) The quantification of MDM2 protein was analyzed according to bands in Figure 4D. (**F**) The quantification of P53 protein was analyzed according to bands in Figure 4D. *p <0.05, **p <0.01, ***p <0.001, n=3 in each group.

Firstly, to further validate the hsa_circ_0002874/miR1273f/MDM2/P53 pathway, miR1273f in A549 cells was downregulated or upregulated via transfection of inhibitor-miR1273f or mimic-miR1273f, and the expression changes of MDM2 and P53 were analyzed by qPCR and Western blot analysis to validate the miR1273f/MDM2/P53 pathway further. As shown in Figure 4A, miR1273f inhibitor was designed to target the binding sites in miR1273f specifically. qPCR results in Figure 4B showed that inhibitor-miR1273f and mimic-miR1273f transfections significantly down- and upregulated the expression of miR1273f to 1/2 (P=0.026) and 250000 times (P=0.011) of that in the negative control group, respectively. Therefore, transfection was successful. As shown in Figure 4C, the expression of MDM2 in A549 cells was upregulated to 1.5 times that in the negative control group (P=0.003) after inhibitor-miR1273f transfection, and mimic-miR1273f transfection significantly downregulated the expression of MDM2 to 2/3 of that in negative control group (P=0.035). As shown in Figure 4D, inhibitor-miR1273f transfection up- and downregulated MDM2 (P=0.044, Figure 4E) and P53 protein expressions (P=0.047, Figure 4F) respectively; whereas mimic-miR1273f transfection down- and upregulated MDM2 (P=0.004, Figure 4E) and P53 expressions (P=0.043, Figure 4F) respectively. The above results indicated that miR1273f was negatively and positively correlated with MDM2 and P53 respectively.

**Figure 5:**
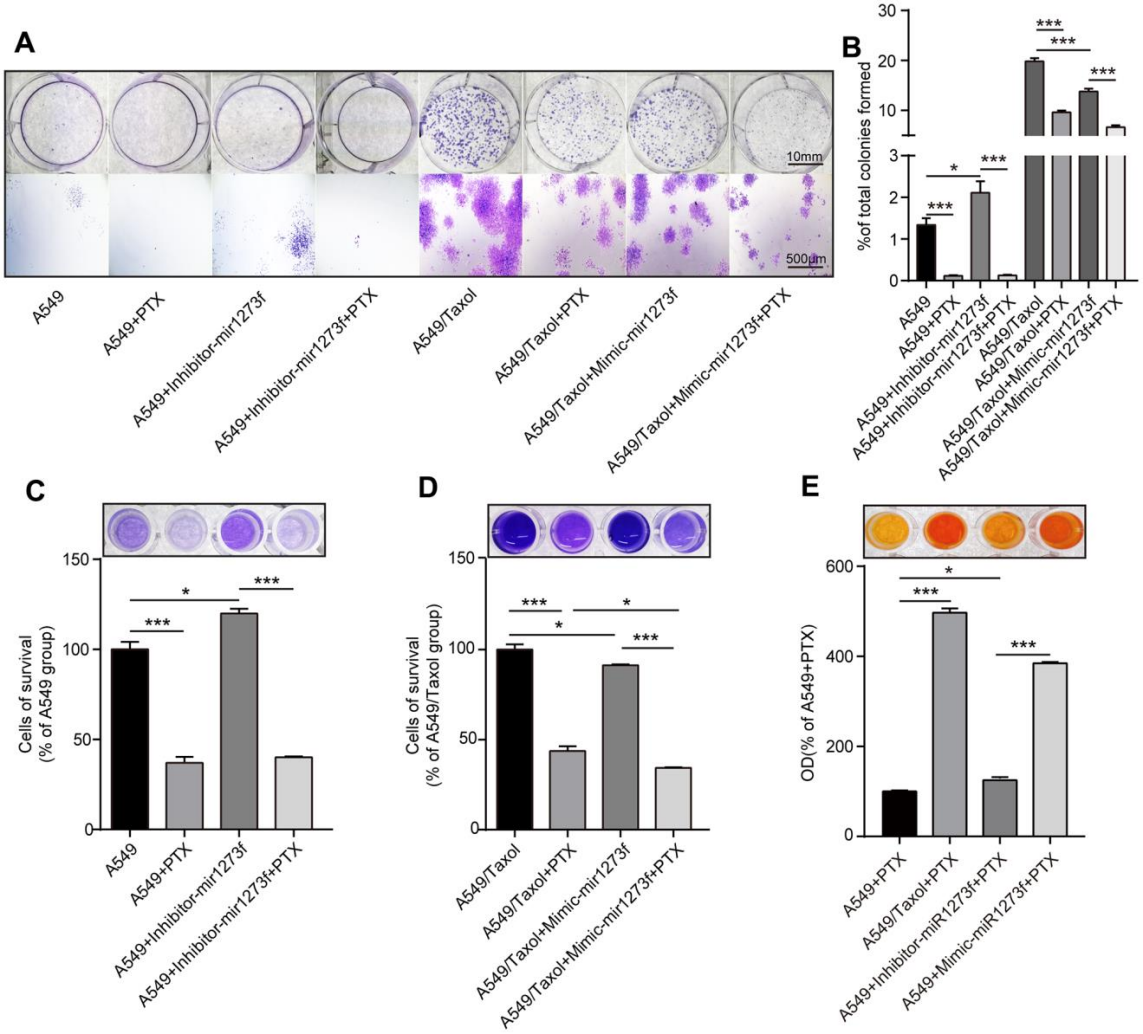
**Knockdown of miR1273f could reduce PTX sensitivity in A549 cell line while overexpression of miR1273f could reverse PTX resistance in A549/Taxol cell line.** (**A**) Cell proliferation ability of A549 and A549/Taxol cells treated with PTX after transfection of inhibitor-miR1273f and mimic-miR1273f was evaluated by colony formation assay and crystal violet staining assay. (**B**) The stained cell area ratio was calculated by 15 random fields per well in Figure 5A (images acquired at 10× magnification). (**C**) OD values of 4 groups with A549 cells were detected at 595 nm using the NanoDrop ND-1000 spectrophotometer. (**D**) OD values of 4 groups with A549/Taxol cells were detected at 595 nm using the NanoDrop ND-1000 spectrophotometer. (**E**) Cell viability of A549 and A549/Taxol cells treated with PTX after transfection of inhibitor-miR1273f and mimic-miR1273f was evaluated by CCK-8 assay. The absorbance at 450 nm of 4 groups was measured using NanoDrop ND-1000 spectrophotometer. *p <0.05, **p <0.01, ***p <0.001, n=3 in each group. Scale bar=10mm (upper) or 500μM (lower).

Secondly, to further analyze the association between hsa_circ_0002874/miR1273f/MDM2/P53 pathway and PTX resistance, we down- and upregulated miR1273f in A549 and A549/Taxol cells via the transfection of inhibitor- and mimic-miR1273f, respectively. Colony formation and CCK-8 assays were then employed to explore the changes in cell proliferation and cell viability in A549 and A549/Taxol cells after transfection. Figure 5A shows the cell proliferation ability of A549 and A549/Taxol cells after the transfection of inhibitor- and mimic-miR1273f via crystal violet staining, respectively. The stained cell area ratio was calculated by 15 random fields per well under 10× magnification. As shown in Figure 5B, inhibitor-miR1273f transfection increased the proliferation of A549 cells (P=0.020); however, this effect was not evident after PTX treatment (P=0.676). Mimic-miR1273f transfection significantly inhibited the proliferation of A549/Taxol cells (P<0.001) and increased the sensitivity of A549/Taxol cells to PTX (P<0.001). After dissolving crystal violet with 10% glacial acetic acid, optical density values were detected at 595 nm using the NanoDrop ND-1000 spectrophotometer. As shown in Figure 5C, inhibitor-miR1273f transfection increased the cellular survival of A549 cells (P=0.015); however, this effect was not evident after PTX treatment (P=0.621). As shown in Figure 5D, mimic-miR1273f transfection significantly inhibited cellular survival of A549/Taxol cells (P=0.046) and increased the sensitivity of A549/Taxol cells to PTX (P=0.028). The above results were consistent with the results shown in Figure 5B. The cell viability of A549 and A549/Taxol cells treated with PTX after the inhibitor's transfection and mimic-miR1273f was evaluated by CCK-8 assay. As shown in Figure 5E, the absorbance at 450 nm of each group indicated that inhibitor- and mimic-miR1273f transfection could attenuate (P=0.011) and strengthen (P<0.001) the cell viability inhibition of PTX in A549 and A549/Taxol cell lines, respectively. The above results indicated that inhibitor- and mimic-miR1273f transfections could reduce and reverse PTX sensitivity and resistance in A549 and A549/Taxol cell lines respectively.

### hsa_circ_0002874 knockdown or miR1273f overexpression could reverse PTX resistance *in vivo*

**Figure 6:**
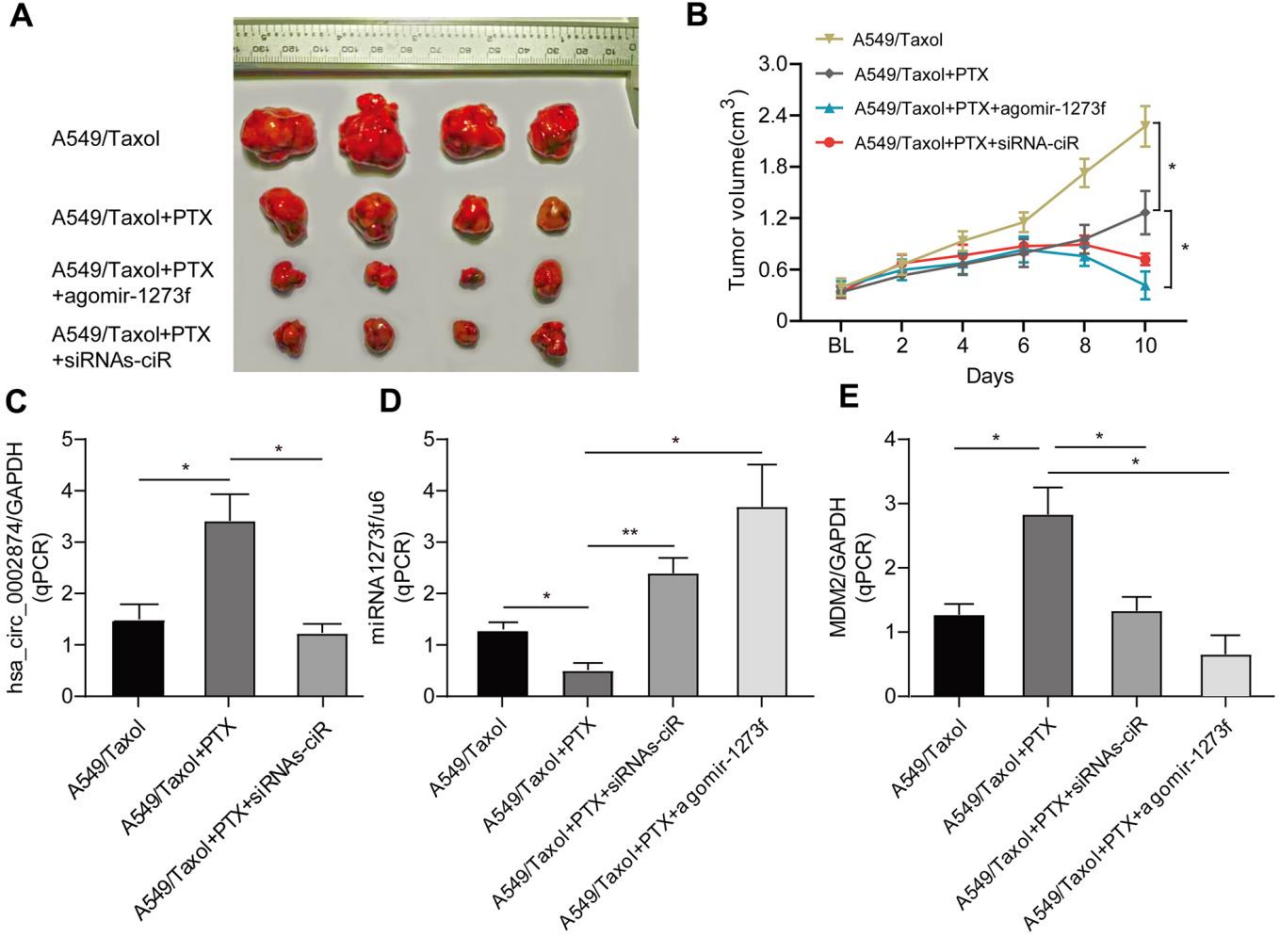
**Effect of agomir-1273f and siRNAs-ciR on tumor growth in drug-resistant xenograft model.** (**A**) The gross morphology of tumors measured on day 10 after injecting agomir-1273f plus PTX, siRNAs-ciR plus PTX or PTX. (**B**) Growth curves of subcutaneous xenograft tumors. Tumor volumes were calculated as length × (square of width) /2. (**C**–**E**) qPCR analysis was performed to detect the expression of hsa_circ_0002874, miR-1273f, and MDM2. BL, baseline tumor volume. *p <0.05, **p <0.01, n=4 in each group.

To determine the important role of hsa_circ_0002874/miR1273f on PTX resistance in LC, we constructed drug-resistant xenografts by subcutaneously injecting A549/Taxol cells. Agomir is a small double-stranded RNA that has been specially labeled and chemically modified. It modulates the biological functions of target genes by simulating endogenous miRNA [16, 17]. As shown in Figure 6A and Figure 6B, the tumor size in the agomir-1273f group and siRNAs-ciR group were significantly smaller than those in the PTX group. The tumor size in the blank group was much larger than those in the PTX group. hsa_circ_0002874, miR1273f, and MDM2 expression levels were also detected by qPCR analysis (Figure 6C–6E). It was observed that the tumor growth was significantly suppressed by intratumorally injecting siRNAs-ciR and agomir-1273f (Figure 6B), with the up- and downregulation of miR1273f (Figure 6D) and MDM2 expressions (Figure 6E) respectively, which validated the effect of hsa_circ_0002874/miR1273f/MDM2/p53 pathway on PTX resistance *in vivo*.

### hsa_circ_0002874 was upregulated in NSCLC tissues and correlated with poor TNM staging

To further validate the role of the hsa_circ_0002874/miR1273f/MDM2/P53 pathway in NSCLC, in addition to the above cellular and molecular experiments, we also collected 20 samples of resected NSCLC tissues. We matched paired non-cancerous tissues from patients diagnosed between September 2018 and May 2019.

**Figure 7:**
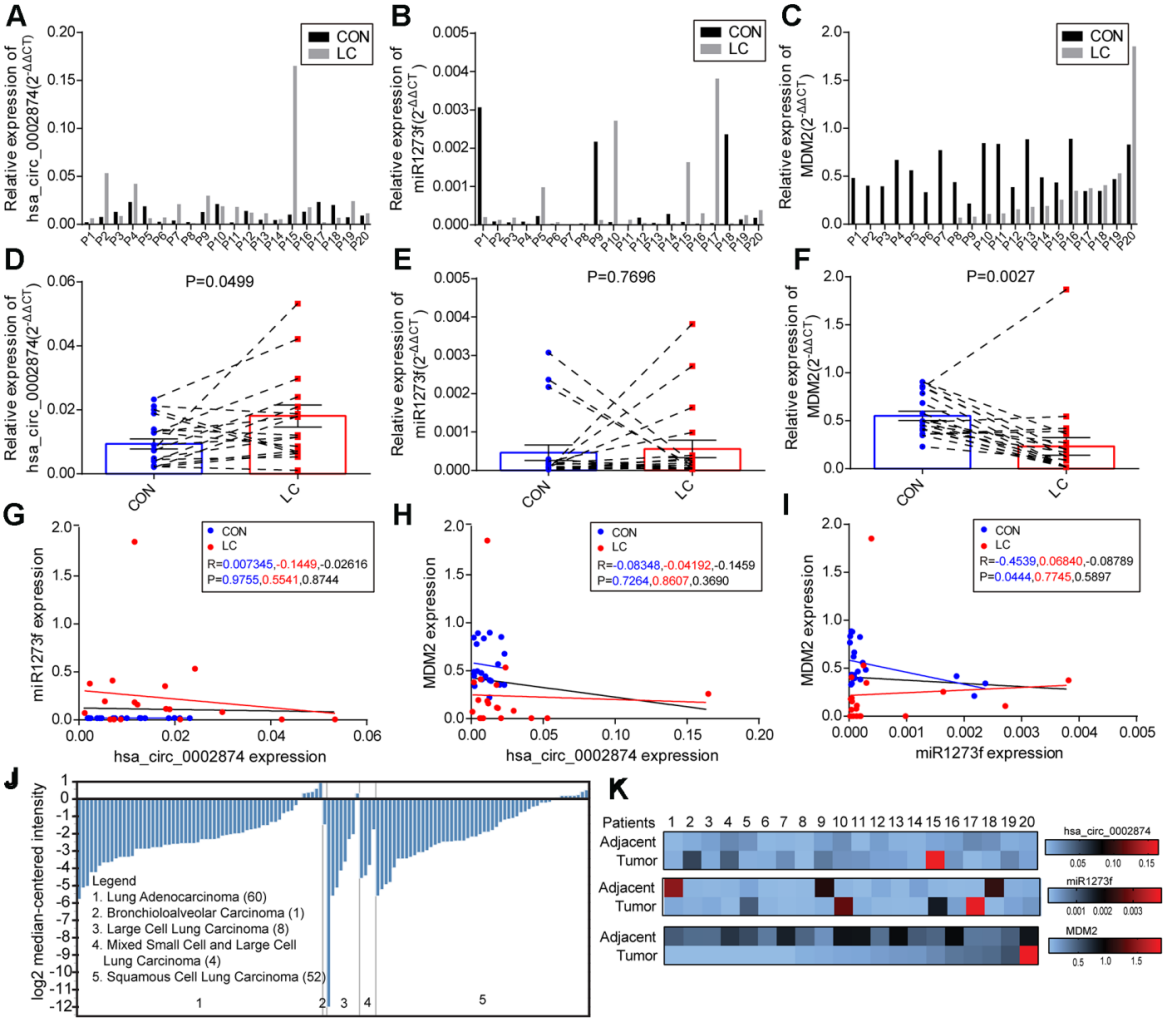
**Levels of hsa_circ_0002874, miR1273f, and MDM2 in samples of NSCLC tissues and paired non-cancerous matched tissues.** (**A**–**C**) Levels of hsa_circ_0002874, miR1273f, and MDM2 were measured using qPCR compared with GAPDH/U6 and calculated using the equation 2-ΔΔCT. (**D**–**F**) Schematic representation of the expression level of hsa_circ_0002874, miR1273f, and MDM2 in 20 NSCLC tissues compared with paired non-cancerous matched tissues. (**G**) Correlation analysis between hsa_circ_0002874 expression and miR1273f expression via Spearman Rank test. (**H**) Correlation analysis between hsa_circ_0002874 expression and MDM2 expression via Spearman Rank test. (**I**) Correlation analysis between miR1273f expression and MDM2 expression via Spearman Rank test. (**J**) Analysis of MDM2 expression in different pathological types of NSCLC via Oncomine database. (**K**) Heat map of the relative expression of hsa_circ_0002874, miR1273f, and MDM2 in 20 patients.

First, qPCR was used to quantify hsa_circ_0002874, miR1273f, and MDM2 mRNA in these 20 paired NSCLC and neighboring non-cancerous tissues (Figure 7A–7C). As shown in Figure 7D, hsa_circ_0002874 was markedly upregulated in NSCLC tissues compared with the respective control (P=0.050). Although there was no significant difference in the amount of miR1273f in cancerous and non-cancerous tissues (P=0.770) (Figure 7E), MDM2 was significantly lower in the tumor (P=0.003) (Figure 7F). A negative relationship between miR1273f and MDM2 was found in paired non-cancerous matched tissues (P=0.044) (Figure 7G). No statistically significant correlation was found between hsa_circ_0002874 and miR1273f in the 20 paired NSCLC and non-cancerous tissues (P=0.874) (Figure 7H), or between hsa_circ_0002874 and MDM2 (P=0.369) (Figure 7I). The Oncomine database was employed to verify the expression of MDM2 in NSCLC tissue, which indicated that levels of MDM2 were decreased in certain pathological types of NSCLC (Figure 7J).

Second, correlations between hsa_circ_0002874/miR1273f/MDM2 expression levels and other clinicopathological parameters in these NSCLC patients were also analyzed. The mean values of hsa_circ_0002874, miR1273f, or MDM2 in NSCLC tissues were used as the cut-off threshold for distinguishing high from low expression groups. It was found that increased expression of hsa_circ_0002874 was clearly related to advanced TNM stage (P=0.045, Table 3). Besides, the amount of hsa_circ_0002874 in tumor tissues was significantly higher than in the adjacent tissues in stage III/IV NSCLC (P=0.048, Table 4). In contrast, for miR1273f, it was found that decreased expression was clearly associated with higher tumor grade (P=0.020, Table 5; P=0.027, Table 6) and advanced T stage (P=0.032, Table 6). Besides, the amount of miR1273f in tumor tissues was significantly lower than in adjacent tissues in node-positive NSCLC (P=0.045, Table 6) and stage III/IV NSCLC (P=0.034, Table 6).

**Table 3:** Analysis of the correlation between expression of hsa_circ_0002874 in LC and its clinicopathological parameters.

**Pathological characteristics**	**Cases (n)**	**hsa_circ_0002874 expression**		**P value**
		High (5)	Low (15)	
**Gender**				0.606
Male	10 (50%)	3 (60%)	7 (46.7%)	
Female	10 (50%)	2 (40%)	8 (56.3%)	
**Age**				0.292
<65	8 (40%)	1 (20%)	7 (46.7%)	
≥65	12 (60%)	4 (80%)	8 (56.3%)	
**Location**				0.210
Left	6 (30%)	2 (40%)	4 (26.7%)	
Right	11 (55%)	1 (20%)	10 (66.7%)	
Others	3 (15%)	2 (40%)	1 (6.7%)	
**T stage**				0.347
1/2	15 (75%)	3 (60%)	12 (80%)	
3/4	2 (10%)	1 (20%)	1 (6.7%)	
Others	3 (15%)	1 (20%)	2 (13.3%)	
**Lymph node metastasis**				0.146
No	9 (45%)	1 (20%)	8 (53.3%)	
Yes	7 (35%)	3 (60%)	4 (26.7%)	
Others	4 (20%)	1 (20%)	3 (20%)	
**Metastasis status**				0.567
No	16 (80%)	4 (80%)	12 (80%)	
Yes	1 (5%)	0	1 (6.7%)	
Others	3 (15%)	1 (20%)	2 (13.3%)	
**TNM stage**				0.045
I/II	12 (60%)	1 (20%)	11 (73.3%)	
III/IV	6 (30%)	3 (60%)	3 (20%)	
Others	2 (10%)	1 (20%)	1 (6.7%)	
**Histology**				0.047
SCC	1 (5%)	1 (20%)	0	
Adenocarcinoma	18 (90%)	3 (60%)	15 (100%)	
Others	1 (5%)	1 (20%)	0	
Tumor grading^*^				0.166
2	2 (10%)	1 (20%)	1 (6.7%)	
3	10 (50%)	1 (20%)	9 (60%)	
Others	8 (40%)	3 (60%)	5 (33.3%)	

^*^ No pathological grading of 1.

**Table 4:** Comparison of hsa_circ_0002874 expression according to patients’ characteristics.

**Pathological characteristics**	**Cases (n)**	**hsa_circ_0002874 in PNT (×10^-2^)**	**P value**	**hsa_circ_0002874 in LC (×10^-2^)**	**P value**	**P value between PNT and LC**
Total	20	1.07±0.74		2.34±3.59		0.132
**Gender**			0.051		0.415	
Male	10	0.76±0.58		3.01±4.97		0.171
Female	10	1.39±0.76		1.66±1.22		0.561
**Age**			0.458		0.283	
<65	8	0.92±0.67		1.25±0.66		0.332
≥65	12	1.18±0.79		3.06±4.53		0.171
**Location**			0.269		0.410	
Left	6	1.34±0.98		1.93±2.23		0.572
Right	11	0.88±0.68		1.32±0.72		0.160
**T stage**			0.530		0.243	
1	8	1.23±0.84		1.24±0.91		0.981
2/3/4	9	0.98±0.76		2.06±1.71		0.101
**Lymph node metastasis**			0.508		0.323	
No	9	1.23±0.95		1.38±1.26		0.779
Yes	7	0.95±0.62		2.13±1.68		0.106
**Metastasis status**			0.000		0.007	
No	16	1.15±0.77		1.74±1.44		0.158
Yes	1	0.21		0.63		-
**TNM stage**			0.635		0.154	
I/II	12	1.14±0.84		1.25±1.11		0.801
III/IV	7	0.96±0.63		2.18±1.64		0.048
**Histology**			0.087		0.000	
SCC	1	0.76		5.32		-
Adenocarcinoma	18	1.09±0.78		1.38±1.05		0.356
Tumor grading^*^			0.009		0.216	
2	2	2.22±0.16		3.04±1.65		0.555
3	10	0.75±0.62		1.51±1.48		0.153

Note: PNT, Paired Noncancerous Tissues. Data were presented as means ± SD. ^*^ No pathological grading of 1.

**Table 5:** Analysis of the correlation between expression of miR1273f in LC and its clinicopathological parameters.

**Pathological characteristics**	**Cases (n)**	**miR1273f expression**		**P value**
		High (4)	Low (16)	
**Gender**				0.264
Male	10 (50%)	1 (25%)	9 (56.3%)	
Female	10 (50%)	3 (75%)	7 (43.8%)	
**Age**				0.068
<65	8 (40%)	0	8 (50%)	
≥65	12 (60%)	4 (100%)	8 (50%)	
**Location**				0.938
Left	6 (30%)	1 (25%)	5 (31.3%)	
Right	11 (55%)	2 (50%)	9 (56.3%)	
Others	3 (15%)	1 (25%)	2 (12.5%)	
**T stage**				0.486
1/2	15 (75%)	3 (75%)	12 (75%)	
3/4	2 (10%)	0	2 (12.5%)	
Others	3 (15%)	1 (25%)	2 (12.5%)	
**Lymph node metastasis**				0.090
No	9 (45%)	3 (75%)	6 (37.5%)	
Yes	7 (35%)	0	7 (43.8%)	
Others	4 (20%)	1 (25%)	3 (18.8%)	
**Metastasis status**				0.633
No	16 (80%)	3 (75%)	13 (81.3%)	
Yes	1 (5%)	0	1 (6.3%)	
Others	3 (15%)	1 (25%)	2 (12.5%)	
**TNM stage**				0.180
I/II	12 (60%)	3 (75%)	9 (56.3%)	
III/IV	6 (30%)	0	6 (37.5%)	
Others	2 (10%)	1 (25%)	1 (6.3%)	
**Histology**				0.656
SCC	1 (5%)	0	1 (6.3%)	
Adenocarcinoma	18 (90%)	3 (75%)	15 (93.8%)	
Others	1 (5%)	1 (25%)	0	
Tumor grading^*^				0.020
2	2 (10%)	1 (25%)	1 (6.3%)	
3	10 (50%)	0	10 (62.5%)	
Others	8 (40%)	3 (75%)	5 (31.3%)	

^*^ No pathological grading of 1.

**Table 6:** Comparison of miR1273f expression according to patients’ characteristics.

**Pathological characteristics**	**Cases (n)**	**miR1273f in PNT (×10^-4^)**	**P value**	**miR1273f in LC (×10^-4^)**	**P value**	**P value between PNT and LC**
**Total**	20	4.62±9.08		5.61±10.19		0.748
**Gender**			0.490		0.281	
Male	10	6.07±11.23		3.09±4.78		0.171
Female	10	3.17±6.58		8.13±13.51		0.561
**Age**			0.279		0.151	
<65	8	7.38±12.35		1.56±0.95		0.205
≥65	12	2.78±6.02		8.31±12.61		0.184
**Location**			0.038		0.663	
Left	6	9.88±13.57		7.12±15.22		0.748
Right	11	0.80±0.74		4.66±7.95		0.124
**T stage**			0.444		0.032	
1	8	7.12±12.02		13.57±15.49		0.396
2/3/4	9	3.31±7.63		1.37±1.22		0.460
**Lymph node metastasis**			0.031		0.174	
No	9	0.66±0.66		9.03±14.00		0.049
Yes	7	11.30±13.40		1.38±0.84		0.045
**Metastasis status**			0.000		0.185	
No	16	3.51±7.51		5.80±10.93		0.496
Yes	1	30.64		2.00		-
**TNM stage**			0.005		0.131	
I/II	12	0.941±0.887		9.58±13.68		0.041
III/IV	7	13.37±13.43		1.18±0.86		0.034
**Histology**			0.082		0.128	
SCC	1	0.90		1.32		-
Adenocarcinoma	18	5.04±9.50		5.25±10.40		0.950
Tumor grading^*^			0.554		0.027	
2	2	0.80±0.07		13.76±18.96		0.436
3	10	5.89±11.33		1.52±1.20		0.241

Note: PNT, Paired Noncancerous Tissues. Data were presented as means ± SD. ^*^ No pathological grading of 1.

Regarding MDM2, the increased expression of MDM2 was evidently related to advanced T stage (P=0.020, Table 7). In summary, the level of MDM2 expression in NSCLC tissues was significantly lower than that in paired non-cancerous matched tissues (P=0.004, Table 8).

**Table 7:** Analysis of the correlation between expression of MDM2 in LC and its clinicopathological parameters.

**Pathological characteristics**	**Cases (n)**	**MDM2 expression**		**P value**
		High (6)	Low (14)	
**Gender**				0.329
Male	10 (50%)	4 (66.7%)	6 (42.9%)	
Female	10 (50%)	2 (33.3%)	8 (57.1%)	
**Age**				0.550
<65	8 (40%)	3 (50%)	5 (35.7%)	
≥65	12 (60%)	3 (50%)	9 (64.3%)	
**Location**				0.394
Left	6 (30%)	2 (33.3%)	4 (28.6%)	
Right	11 (55%)	3 (50%)	8 (57.1%)	
Others	3 (15%)	1 (16.7%)	2 (14.3%)	
**T stage**				0.020
1/2	15 (75%)	3 (50%)	12 (85.7%)	
3/4	2 (10%)	2 (33.3%)	0	
Others	3 (15%)	1 (16.7%)	2 (14.3%)	
**Lymph node metastasis**				0.771
No	9 (45%)	2 (33.3%)	7 (50%)	
Yes	7 (35%)	2 (33.3%)	5 (35.8%)	
Others	4 (20%)	2 (33.3%)	2 (14.3%)	
**Metastasis status**				0.506
No	16 (80%)	5 (83.3%)	11 (78.6%)	
Yes	1 (5%)	0	1 (7.1%)	
Others	3 (15%)	1 (16.7%)	2 (14.3%)	
**TNM stage**				0.710
I/II	12 (60%)	3 (50%)	9 (64.3%)	
III/IV	6 (30%)	2 (33.3%)	4 (28.6%)	
Others	2 (10%)	1 (16.7%)	1 (7.1%)	
**Histology**				0.539
SCC	1 (5%)	0	1 (7.1%)	
Adenocarcinoma	18 (90%)	5 (83.3%)	13 (92.9%)	
Others	1 (5%)	1 (16.7%)	0	
Tumor grading^*^				0.273
2	2 (10%)	0	2 (14.3%)	
3	10 (50%)	4 (66.7%)	6 (42.9%)	
Others	8 (40%)	2 (33.3%)	6 (42.9%)	

^*^ No pathological grading of 1.

**Table 8:** Comparison of MDM2 expression according to patients’ characteristics.

**Pathological characteristics**	**Cases (n)**	**MDM2 in PNT (×10^-1^)**	**P value**	**MDM2 in LC (×10^-1^)**	**P value**	**P value between PNT and LC**
**Total**	20	5.50±0.21		0.23±0.41		0.004
**Gender**			0.275		0.346	
Male	10	4.96±1.81		3.22±5.70		0.370
Female	10	6.05±2.43		1.43±1.35		0.000
**Age**			0.587		0.740	
<65	8	5.17±2.21		1.94±2.07		0.009
≥65	12	5.73±2.22		2.59±5.16		0.049
**Location**			0.029		0.775	
Left	6	4.54±1.23		1.62±1.92		0.010
Right	11	6.59±2.20		1.34±1.76		0.000
**T stage**			0.972		0.392	
1	8	5.69±2.72		1.36±1.52		0.002
2/3/4	9	5.73±2.02		3.30±6.04		0.269
**Lymph node metastasis**			0.023		0.888	
No	9	6.44±2.33		1.32±1.43		0.000
Yes	7	4.39±1.71		1.45±2.25		0.017
**Metastasis status**			0.125		0.044	
No	16	5.76±2.36		2.53±4.6		0.018
Yes	1	4.80		0.00		-
**TNM stage**			0.015		0.627	
I/II	12	6.46±2.22		2.69±5.15		0.036
III/IV	7	3.82±0.97		1.67±2.16		0.046
**Histology**			0.006		0.029	
SCC	1	4.00		0.00		-
Adenocarcinoma	18	5.65±2.24		2.44±4.33		0.009
Tumor grading^*^			0.305		0.605	
2	2	7.57±2.29		0.53±0.75		0.020
3	10	5.72±2.29		2.79±5.73		0.151

Note: PNT, Paired Noncancerous Tissues. Data were presented as means ± SD. ^*^ No pathological grading of 1.

**Figure 8:**
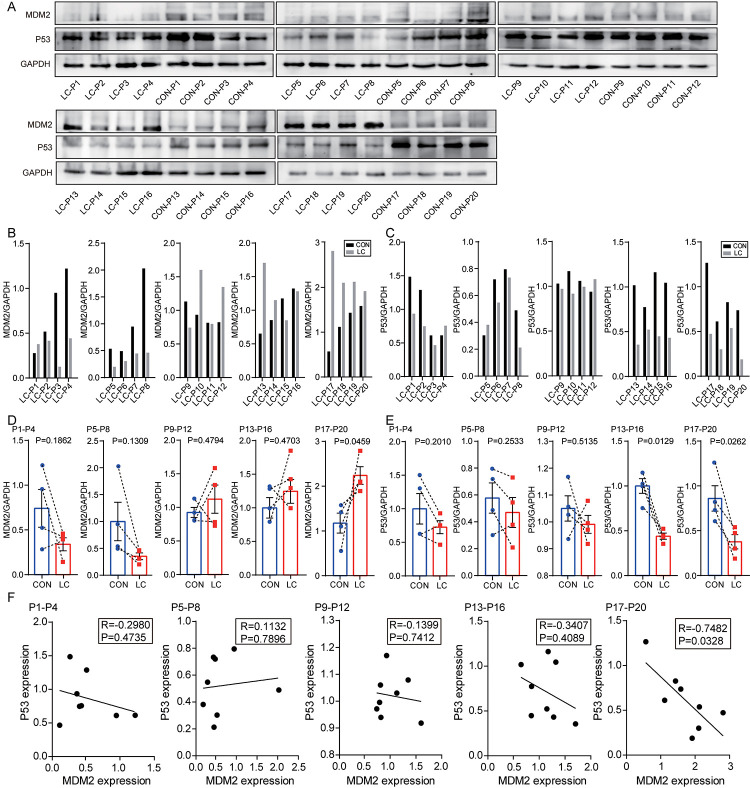
**Levels of MDM2 and P53 protein in samples of NSCLC tissues and paired non-cancerous matched tissues.** (**A**) MDM2 and P53 expression in 20 NSCLC tissues compared with paired non-cancerous matched tissues were determined by western blot. (**B**) The quantification of MDM2 protein was analyzed according to bands in Figure 8A. (**C**) The quantification of P53 protein was analyzed according to bands in Figure 8A. (**D**, **E**) Schematic representation of the expression level of MDM2 and P53 in 20 NSCLC tissues compared with paired non-cancerous matched tissues. (**F**) Correlation analysis between MDM2 expression and P53 expression via Spearman Rank test.

Third, based on the qPCR results in Figure 7C, 20 paired NSCLC and paracancerous tissues were divided into 5 groups according to the sequence of MDM2 expression from low to high for western blotting experiments (Figure 8A). The levels of MDM2 and P53 protein are shown in Figures 8B, 8C, respectively. As shown in Figure 8D, MDM2 was markedly upregulated in NSCLC tissues compared with paracancerous tissues in MDM2 high-expression group (P=0.046). However, no significant difference was found between NSCLC and paracancerous tissues in the 4 relatively low-MDM2 expression groups (P=0.186, 0.131, 0.479, 0.470). As shown in Figure 8E, P53 was markedly downregulated in NSCLC tissues compared with that in paracancerous tissues in the two high-MDM2 expression groups (P=0.013, 0.026). However, no significant difference was found between NSCLC and paracancerous tissues in the 3 relatively low-MDM2 expression groups (P=0.201, 0.253, 0.514). A negative relationship between MDM2 and P53 was found in high-MDM2 expression group (R=-0.748, P=0.033; Figure 8F). No statistically significant correlation was found in another 4 relatively low-MDM2 expression groups between MDM2 and P53 (P=0.474, 0.790, 0.741, 0.409). The above results indicated that MDM2 could be associated with the progression of NSCLC in the high-MDM2 expression group, which was negatively correlated with P53.

## DISCUSSION

The focus of this study was to determine whether the expression of hsa_circ_0002874 in NSCLC was aberrant and to elucidate molecular mechanisms influencing apoptosis and PTX resistance. Our results suggest that PTX exerted its effects in the A549 cell line by down-regulating the expression of hsa_circ_0002874, which in turn could regulate the expression of MDM2 and P53 via acting as a sponge for miR1273f. High expression of hsa_circ_0002874 is associated with PTX resistance in NSCLC cells, and downregulation of hsa_circ_0002874 or upregulation of miR1273f could increase the chemosensitivity of A549/Taxol to PTX in xenograft models. According to our analysis of NSCLC and paired matched non-cancerous tissues, hsa_circ_0002874 was upregulated in NSCLC and correlated with poor TNM staging. These findings offer a new vista for research into the role of circular RNA in the development of NSCLC, and provide a new perspective for analyzing PTX resistance in this cancer. This study also explored the use of siRNAs-ciRs or mimic-miRs as new approaches to reverse PTX resistance.

The previous research of our group found that PTX depends on P53 protein to promote the apoptosis of A549 cells and can up-regulate P53 protein expression via up-regulating the level of long-chain ncRNA MEG3 [18]. Association studies between PTX and ncRNA non-coding RNA have often focused on PTX resistance. In terms of miRNAs, ursolic acid has recently been reported to reverse the chemical resistance of PTX to breast cancer cells by targeting miRNA-149-5p [19]. MiR-155-3p can also act as a tumor suppressor and reverse PTX resistance via the negative regulation of MYD88 in human breast cancer [20].

In studies of lncRNAs, it was recently reported that knockdown of LINC00511 could reduce the resistance of cervical cancer cells to PTX [21]. However, very few studies have examined associations between circRNAs and PTX resistance. Previous bioinformatics analysis suggested that 2909 significantly upregulated and 8372 downregulated circRNAs were detectable in A549/Taxol relative to parental A549 cells [22], leaving many unexplored areas for the roles of circular RNA in NSCLC. In our research, the expression of hsa_circ_0002874 was found to be downregulated by PTX in A549 cells, which could represent a new mechanism of action of PTX. Moreover, our results indicated that strong expression of hsa_circ_0002874 was associated with NSCLC PTX resistance. To explore methods to reverse the PTX resistance of NSCLC cells, transfection of inhibitor-miR1273f and mimic-miR1273f was performed. The results showed that knockdown of miR1273f could reduce PTX sensitivity in A549 cells while overexpression of miR1273f reversed PTX resistance in the A549/Taxol cell line.

An increased understanding of the biological roles of circRNA has resulted in questions on the relationship between circRNA and NSCLC becoming a hot topic. In 2017, some scholars analyzed the circRNA expression profile of patients with early lung adenocarcinoma [23]. A total of 357 dysregulated circRNAs were found in tumor samples, suggesting their potential role in lung cancer. Abnormal expression of circRNA clusters in early lung adenocarcinoma could provide potential targets for early diagnosis of the disease. More recently, many other circRNAs have been reported to be related to NSCLC. The expression of circ_0067934 was significantly increased in NSCLC tissues and was associated with poor prognosis of NSCLC. Silencing circ_0067934 inhibited proliferation, migration, and invasion of NSCLC cells [24]. NSCLC tumor specimens exhibited higher levels of circP4HB than paired healthy lung samples, which would promote NSCLC invasion and metastasis by adsorption of miR-133a-5p [25]. In our research, hsa_circ_0002874 was found to be strongly upregulated in NSCLC tissues compared to paired non-cancerous matched tissues.

In our study, the level of miR1273f, a downstream target of hsa_circ_0002874, did not differ significantly between NSCLC tissues and paired non-cancerous matched tissues. However, this could be due to the small sample size in this pilot study, with only 20 pairs of samples, or other pathways affecting the expression of miR1273f. There are many studies in the literature on the relationship between miRNA and NSCLC. A recent report suggested that miRNA-621 is closely related to the pathological grade and poor prognosis of NSCLC. Furthermore, miRNA-621 could inhibit the malignant progression of NSCLC by modulating SIX4 expression [26]. miRNA could also be used as a candidate biomarker for early diagnosis of NSCLC. Recently, it has been reported that miRNA-23a and miRNA-451 may be useful as potential biomarkers for early diagnosis of NSCLC, and both together may be more effective for diagnosis than either alone [27]. MiRNA-17 and miRNA-222 can also be considered as non-invasive biomarkers for detecting early lung cancer development and metastasis in patients with NSCLC [28].

Regarding the relationship between MDM2 protein and NSCLC tissues, MDM2 is highly expressed as a proto-oncogene in cancer tissues. MDM2 is significantly upregulated in lung adenocarcinoma tissues compared with adjacent tissues [29]. However, MDM2 expression is not significantly different between cancer and paracancerous tissues in patients with NSCLC according to Western blot analysis results [30]. In our research, although the increased expression of MDM2 was evidently related to advanced T stage, the level of MDM2 expression in NSCLC tissues was significantly lower than that in paired non-cancerous matched tissues. The Oncomine database analysis indicated the low expression of MDM2 in different pathological subgroups. The above results showed that the expression of MDM2 in NSCLC remains uncertain. Besides, the contingency effect caused by the small sample size was also an interference factor.

Regarding the relationship between P53 protein and NSCLC tissues, the most common mutated gene in lung adenocarcinoma and lung squamous cell carcinoma is p53, found in 45%-70% of adenocarcinomas and 60%-80% of squamous cells cancer [31]. Due to the high mutation rate of p53 in NSCLC, targeting mutated p53 and restoring its wild-type function is a potential therapeutic strategy and is used to develop new compounds to treat cancer. Nutlin, for example, is a compound capable of increasing wild-type p53’s anti-tumor activity by blocking the interaction between p53 and MDM2 *in vivo* (E3 ubiquitin ligase of p53) [32, 33]. Mammalian cell lines and mouse xenograft models show that PRIMA (p53 reactivation and induction of massive apoptosis) can bind to and convert mutant p53 to its wild-type structure, leading to growth inhibition and apoptosis [34, 35]. RETRA (reactivation of transcriptional reporter activity) is another compound which inhibits mutant p53 activity by releasing p73 (p53 family protein with a high level of sequence similarity) from the p53 complex and activating target proteins associated with growth inhibition and apoptosis induction [36, 37]. Hence, developing and discovering new molecules targeting abnormal p53 or promoting the pro-apoptotic role of wild-type p53 can aid clinical cancer therapy [38]. This research is dedicated to developing and discovering new molecules that enhance the pro-apoptotic effect of wild-type p53 via hsa_circ_0002874/miR1273f/MDM2/P53 pathway.

### Limitations

There are some limitations to our analysis that deserve discussion. First, the mechanism study was carried out in a single pair of cell lines A549 and A549/Taxol, and more studies on other cell lines need to be further studied. Second, the mechanism of paclitaxel treatment regulating the expression of hsa_circ_0002874 needs further study. Third, only 20 NSCLC tissues and paired non-cancerous matched tissues were available for study. Limited sample size weakens conclusions on the abnormal expression of hsa_circ_0002874 in NSCLC. Forth, also due to the small sample size, there are many false positives in the chi-square test and the Student t-test in Table 3–8.

## CONCLUSION

CircRNA hsa_circ_0002874 is strongly expressed in NSCLC tissues and maybe a potential marker of PTX resistance. CircRNA hsa_circ_0002874 acts as a sponge for miR1273f and thereby affects the level of MDM2, eventually acting as a tumor promoter in NSCLC.

## MATERIALS AND METHODS

### Reagents and antibodies

PTX was purchased from Aladdin. Dulbecco's Modified Eagle Medium (DMEM) /F12 was purchased from Gibco. Fetal bovine serum (FBS) was purchased from Biological Industries. Trypsin, crystal violet, 3- (4,5-dimethylthiazol-2-yl) -2,5-diphenyltetrazolium bromide (MTT) kit, Cell Counting Kit-8, and P53 antibody were purchased from Beyotime Biotechnology. Trizol was purchased from Ambion. RevertAid First Strand complementary DNA (cDNA) Synthesis Kit was purchased from Thermo. SYBR Green Polymerase Chain Reaction (PCR) kit was purchased from Biomake. Glyceraldehyde 3-phosphate dehydrogenase (GAPDH) antibody was purchased from Signalway Antibody. Goat anti-mouse IgG horseradish peroxidase horseradish peroxidase (HRP) -conjugated secondary antibody was purchased from Santa Cruz Biotecnology. MDM2 antibody was purchased from Affinity. Polyvinylidene difluoride (PVDF) membrane was purchased from Immobilon. 0.3% triton-X was purchased from Vetec. Lipofectamine 3000 transfection reagent was purchased from Invitrogen. Mimic-1273f and Inhibitor-1273f were synthesized by GenePharma (Shanghai, China). siRNAs-ciR and pCD25-ciR were synthesized by Geneseed (Guangzhou, China). The siRNAs-ciR used in this study is a mixture of 3 types of siRNA, and their sequences are 5’-AATCCTGGGAAAGGCTTAT-3’, 5’-ATCCTGGGA AAGGCTTATA-3’, and 5’-CTGGGAAAGGCTTATAACC-3’. Dual luciferase reporter vector plasmid (miR1273f) was purchased by GenePharma (Shanghai, China). Dual luciferase reporter gene fluorescence detection kit was purchased by Promega. Agomir-1273f (catalog number: B06002) was purchased by GenePharma (Shanghai, China).

### Tissue specimens

A total of 20 NSCLC tissues and paired non-cancerous matched tissues were collected through surgical resection from patients diagnosed between September 2018 and May 2019 at the The Second Affiliated Hospital of Suzhou University (Suzhou, Jiangsu, China). With the guidance of a skillful pathologist, we collected normal lung samples with a distance of ≥2 cm from the edge of cancer tissue. All patients did not receive radiotherapy and chemotherapy before surgery. All specimens were collected under the guidance of the HIPAA protocol. The study was approved by the Ethics Committee of Second Affiliated Hospital of Suzhou University, and written informed consent was obtained from all the patients. TNM stage classification complied with the NCCN Clinical Practice Guidelines in Oncology: Non-Small Cell Lung Cancer (Version 2.2019).

### Cell lines and cell culture

Human A549 cell lines were supplied by the Cell Bank of Type Culture Collection of the Chinese Academy of Sciences, Shanghai, China (CBP60084). Human A549/Taxol cell lines were purchased from Yaji Biotechnology Company, Shanghai, China (YS421C). A549 cells were cultured in DMEM/F12 medium supplemented with 10% fetal bovine serum (which contained 100 U/ml penicillin and 100 mg/ml streptomycin). A549/Taxol cells were cultured in DMEM/F12 medium supplemented with 10% fetal bovine serum (which contained 5μM PTX, 100 U/mL penicillin, and 100 mg/mL streptomycin).

### CircRNA and miRNA screening

Primers for 18 circRNAs resistant to breast cancer cell MCF-7 doxorubicin [14] were designed, and qPCR was used to screen for the most stable and robust circRNA expression after PTX administration. Subsequently, it was submitted to Suzhou Jima Gene Company for technical evaluation to ensure that the length of the selected circRNA can ensure the synthesis of high-quality pCDNA and siRNAs for subsequent experimental steps. The 18 primary screening circRNAs were shown in Table 9.

**Table 9:** 18 circRNAs^*^ for primary screening.

**CircRNA ID**	**CircRNA type**	**Chrom**
Upregulated circRNAs		
hsa_circ_ 0002113	exonic	Chr21
hsa_circ_ 0001667	exonic	Chr7
hsa_circ_ 0006528	exonic	Chr5
hsa_circ_ 0002874	exonic	Chr9
hsa_circ_ 0002168	exonic	Chr20
hsa_circ_ 0086241	exonic	Chr9
hsa_circ_ 0007769	exonic	Chr6
hsa_circ_ 0092276	intronic	Chr3
hsa_circ_ 0044556	exonic	Chr17
hsa_circ_ 0003183	exonic	Chr8
hsa_circ_ 0085567	exonic	Chr8
hsa_circ_ 0085495	exonic	Chr8
Downregulated circRNAs		
hsa_circ_ 0008131	exonic	ChrX
hsa_circ_ 0003838	exonic	Chr15
hsa_circ_ 0007551	exonic	Chr5
hsa_circ_ 0005004	exonic	Chr7
hsa_circ_ 0006903	exonic	Chr12
hsa_circ_ 0018293	exonic	Chr10

^*^ According to Screening circular RNA related to chemotherapeutic resistance in breast cancer [14].

Hsa_circ_0002874 was screened and its target miRNAs predicted by circMir 1.0, RegRNA 2.0 and MirTrap software were hsa-miR-1273f, hsa-miR-4726-5p, hsa-miR-2115-5p, and hsa-miR-4649-5p. The literature review, miRBase and TargetScan web analysis were used to predict the target genes of the four, and the downregulated protein expressions were: MDM2, SHP-1, Erbb2, MLLT6. The expressions of predicted miRNAs after the administration of PTX were verified by qPCR to estimate the true target miRNA of hsa_circ_0002874 (Table 2).

### RNA extraction and quantitative polymerase chain reaction (qPCR) analysis

According to the manufacturer's protocol, total RNA was extracted with Trizol reagent. According to OD260/280 readings, the purity and concentration of RNA were determined by NanoDrop ND-1000 spectrophotometer. Total RNA (500ng) was reverse transcribed into cDNA with a final volume of 20 μl. RevertAid First Strand cDNA Synthesis Kit (Thermo) was used under standard conditions with random primers and oligo dT primers. Purity and concentration of DNA were determined by NanoDrop ND-1000 spectrophotometer. Then, the SYBR Green PCR kit was used for qPCR. The reaction was set as follows: 94° C for 3 min, 30 cycles at 94° C for 30 s, 55° C for 30 s, and 72° C for 30 s. Final extension was performed at 72° C for 7 min. The results of qPCR normalized to the expression of GAPDH. The results of qPCR were analyzed relative to the threshold cycle (Ct) value and converted into multiple values according to the rule of 2^-ΔΔCT^. The primers used are shown in Table 1.

### MTT assay

The cells were seeded into a 96-well plate (Corning) at a density of 5×10^3^ cells/well in 200 μl culture medium. After treatment, the cells were incubated in 200 ml DMEM/F12 containing 0.5 mg/ml MTT at 37° C for 4 hours. Afterward, the supernatant was removed, and the cells were lysed in 200 μl dimethyl sulfoxide (DMSO) for 10 min at 37° C. Optical density (OD) values were detected at 490 nm. The obtained values were presented as folds of the control group.

### Western blot analysis

Western blot analysis was performed using standard procedures. Briefly, total protein was extracted and isolated by 10% sodium dodecyl sulfate polyacrylamide gel electrophoresis (SDS-PAGE) and transferred to a PVDF membrane. To block non-specifically bound, the membrane was incubated with 5% skim milk powder for 1 hour at room temperature. Membranes were then incubated with primary antibody against MDM2 or P53 (1:1000) followed by HRP labeled secondary antibody and detected by chemiluminescence. An anti-GAPDH antibody (1:1000) was used as a protein loading control.

### Dual-luciferase reporter assay

MDM2 3’ UTR was amplified from cDNA of 293 cells and inserted into pGL-3 (Promega, USA). The 293 cells (GenePharma, Shanghai, China) were cotransfected with the wild-type 3’UTR of MDM2 containing the putative miR1273f binding site (Site 1: 2709-2715) and mutant MDM2 3’ UTR with either NC mimics or miR1273f mimics via Lipofectamine 3000. After transfection, the cells were cultivated at 37° C, 5% CO2 for 4 h. Then, the luciferase activities were confirmed using a dual-luciferase reporter assay system according to the manufacturer’s protocol.

### Transfection

A549 cells were seeded into 6-well plates, incubated overnight and transfected with siRNAs-ciR/pCD25-ciR plasmid or miR1273f inhibitor/negative control. A549/Taxol cells were transfected with the miR1273f mimic/negative control under the same conditions. The sequences used for transfection were listed in Table 10. Lipofectamine 3000 was used as a transfection reagent according to the manufacturer's recommendations. After transfection for 48 hours, cells were used for functional analysis.

**Table 10:** Transfected gene sequences.

**Gene**	**Sequence (5'→3')**
hsa_circ_0002874-siRNAs	AATCCTGGGAAAGGCTTAT
	ATCCTGGGA AAGGCTTATA
	CTGGGAAAGGCTTATAACC
Negative control (NC)	Sense UUCUCCGAACGUGUCACGUTT
	Antisense ACGUGACACGUUCGGAGAATT
miR-1273f mimic	Sense GGAGAUGGAGGUUGCAGUG
	Antisense CUGCAACCUCCAUCUCCUU
miRNA inhibitor NC	CAGUACUUUUGUGUAGUACAA
miR-1273f inhibitor	CACUGCAACCUCCAUCUCC

### Colony formation assay

Transfected A549 or A549/Taxol cells were seeded in 6-well plates at 5×10^3^ cells per well. After incubation for 36 hours at 37° C in a 5% CO_2_ humidified incubator, the cells were incubated with medium supplemented with PTX (2μM) and cultured at 37° C in a 5% CO_2_ humidified incubator for 7 days. After colony formation was observed, the medium was removed. The cells were washed twice with phosphate buffered saline (PBS), fixed with 4% formaldehyde for 10 minutes, and stained with 5% crystal violet for 10 minutes. The stained cell area ratio was calculated by randomly photographing 15 fields per well under a 10× microscope. Finally, after dissolving crystal violet with 10% glacial acetic acid, OD values were detected at 595 nm. The obtained values were presented as folds of the control group.

### CCK8 assay

After 48 hours of transfection in 96-well plates, the freshly prepared medium contained PTX at a final concentration of 10μM. The medium was added to the wells with 7 replicate wells per set. After 48 hours of incubation, cell viability was measured using CCK-8 kit according to the manufacturer's instructions. The absorbance at 450 nm was measured using NanoDrop ND-1000 spectrophotometer.

### Xenograft assay

All experimental protocols were approved by the Animal Ethics Committee of Second Affiliated Hospital of Soochow University. A total of 20 BALB/c nude mice (4 weeks old) weighing 20.75±1.2g were fed a pellet diet and housed under controlled environment with a temperature of 24±2 C and air humidity of 60±2%. For the drug-resistant xenograft model, A549/Taxol cells were subcutaneously injected into the armpits of nude mice (1×10^6^ cells per animal). From the 10th day after cell injection, the engraftment of tumor was confirmed and the baseline tumor size was evaluated. The xenograft-bearing mouse models were randomized into four groups (n=5), five mice were were intraperitoneally injected with PTX (15mg/kg each time) and intratumorally injected with agomir-1273f (5 nmol each time); five mice were intraperitoneally injected with PTX and intratumorally injected with siRNAs-ciR; five mice were intraperitoneally injected with PTX and intratumorally injected with PBS; and the remaining five mice were intratumorally and intraperitoneally injected with PBS as a control once every 3 days. Tumor formations were monitored by measuring the length (L) and width (W) with calipers every 2 days, and the volumes were calculated using the following formula: (L×W×W)/2. All mice were sacrificed on 10 days, and the tumors were neatly excised. Tumor tissues were then subjected to RNA isolation for qPCR analysis.

### Statistical analysis

All statistical analyses were performed using SPSS 22.0 software (IBM) and Graph pad Prism 5.0. Differences between NSCLC tissues and paired non-cancerous matched tissues were analyzed using the Student’s t test. One-way ANOVA further analyzed the correlations between hsa_circ_0002874 expression levels and clinicopathological factors. The correlations among hsa_circ_0002874 expression, miR1273f expression, and MDM2 expression were explored by Pearson correlation analysis. P <0.05 was considered statistically significant.
